# Sugar Treatments Can Induce *AcLEAFY COTYLEDON1* Expression and Trigger the Accumulation of Storage Products during Prothallus Development of *Adiantum capillus-veneris*

**DOI:** 10.3389/fpls.2017.00541

**Published:** 2017-04-21

**Authors:** Yu-Han Fang, Xia Li, Shu-Nong Bai, Guang-Yuan Rao

**Affiliations:** ^1^College of Life Sciences, Peking UniversityBeijing, China; ^2^RDFZ XiShan SchoolBeijing, China

**Keywords:** *Adiantum capillus-veneris*, *LEAFY COTYLEDON1*, seed maturation process, sugar treatments, prothallus development

## Abstract

A seed is an intricate structure. Of the two development processes involved in seed formation, seed maturation, or seed program includes accumulation of storage products, acquisition of desiccation tolerance, and induction of dormancy. Little is known about how these processes were originated and integrated into the life cycle of seed plants. While previous investigation on seed origin was almost exclusively through fossil comparison in paleobotany, a wealth of information about the key role of *LEAFY COTYLEDON1* (*LEC1*) in seed formation of spermatophyte inspired a new approach to investigating the seed origin mystery. Here, we examined the expression pattern of *AcLEC1* during the entire life cycle of *Adiantum capillus-veneris*, a non-seed plant, confirmed no *AcLEC1* gene expression detectable in prothalli, demonstrated inductive expressed by both sucrose and glucose in prothalli. As expected, we found that sugar treatments delayed prothallus development, promoted differentiation of reproductive organs, and triggered accumulation of storage products. These findings demonstrated links between the sugar treatments and the induction of *AcLEC1* expression, as well as the sugar treatments and the events such as accumulation of storage products, which is similar to those considered as seed maturation process in seed plants. These links support a modified hypothesis that inductive expression of *LEC1* homologs during embryogenesis might be a key innovation for the origin of the seed program.

## Introduction

The seed habit represents the most successful innovation in land plant sexual reproduction (Linkies et al., 2010). It not only contributes to the remarkable prosperity of spermatophytes, but also serves as essential food for humans (Kenrick and Crane, 1997; Becker and Marin, 2009; Radoeva and Weijers, 2014). The seed is an intricate structure comprising of an early embryo derived from a zygote, the seed coat derived from integuments, and extraembryonic tissues. Two developmental processes are involved in the seed formation: one is morphogenesis, through which, cells derived from zygote division are organized as a particular structure called embryo which will further elaborates into sporophyte (Goldberg et al., 1994; Harada, 1997; Gutierrez et al., 2007). Another is called seed maturation or seed program, of which, three physiological and biochemical processes are included, i.e., accumulation of storage products, acquisition of desiccation tolerance, and induction of dormancy (Harada, 1997; Sreenivasulu and Wobus, 2013). While all land plants are embryophyta, i.e., plants with embryos, the seed maturation is unique for spermatophytes (Goldberg et al., 1994; Harada, 1997; Vicente-Carbajosa and Carbonero, 2005). This process generally starts from heart stage embryo, superposing over the embryogenesis, and ends as a mature seed in which the morphogenesis of the embryo was repressed (Harada, 1997). When a favorable environment comes, the dormancy is broken, the reserves are consumed and morphogenesis of the embryo resumes. It is generally considered that the seed maturation enables embryos of spermatophytes to better endure harsh environments and get more chances of dispersal, and therefore benefits the prosperity of seed-bearing plants on land territory (Becker and Marin, 2009; Pires and Dolan, 2012). However, little is known about how such a process is originated and integrated into the life cycle of seed plants.

Previous studies on seed origin was almost exclusively through fossil comparison in paleobotany (Taylor and Taylor, 1993; Niklas, 1997; Doyle, 2006). However, identification of an *Arabidopsis* mutant *leafy cotyledon 1* (*lec1*) opened up a window to peer the secret of seed origin (Meinke, 1992). While the function of *LEAFY COTYLEDON1* (*LEC1*) gene was first considered as taking responsible for homeotic transition from cotyledons to true leaves (Meinke, 1992), later investigations indicated it serving as a master regulator that coordinates many facets of seed maturation (Meinke et al., 1994; West et al., 1994).

*LEC1* gene encodes a LEC1-type *HAP3* subunit of the CCAAT-binding transcription factor. Overexpression of *LEC1* leads to suppression of shoot development regeneration and induction of somatic embryos (Lotan et al., 1998; Casson and Lindsey, 2006; Junker et al., 2012). Moreover, LEC1 protein is required for proper expression of genes involved in seed maturation (Kwong et al., 2003; Lee et al., 2003; Braybrook and Harada, 2008). According to Harada (1997), the seed maturation is an intrusive process into embryogenesis. If it was the case, the master regulator *LEC1* gene should be an ideal subject to test a hypothesis that seed maturation process should be emerged along with the origin of *LEC1*.

Yang et al. (2005) and Xie et al. (2008) have conducted systematic analysis of *LEC1* related *HAP3* genes among a wide range of species covering green algae, bryophytes, pteridophytes, and spermatophytes. They found that these genes could be classified into LEC1-type and non-LEC1-type. While LEC1-type *HAP3* gene exists in all spermatophytes but not in green algae and bryophytes as anticipated, it was unexpected that such genes were identified in pteridophytes, including lycophytes *Selaginella sinensis* and *Selaginella davidii*, as well as fern *Adiantum capillus-veneris* (Xie et al., 2008; Kirkbride et al., 2013). The LEC1-type *HAP3 SsLEC1, SdLEC1*, and *AcLEC1* can complement the *lec1* mutant phenotype of *Arabidopsis* and expressed upon drought and ABA stress (Xie et al., 2008). These findings suggest that pteridophytes LEC1-type *HAP3* genes are not pseudo- but functional genes. Although no LEC1-type *HAP3* gene found in green algae and bryophytes supports the hypothesis that seed maturation process is emerged along with the origin of *LEC1* gene, existence of functional LEC1-type *HAP3* genes in pteridophytes is contradict to the hypothesis.

Parallel to the above mentioned gene sequence and function analysis, Li et al. (2013) have developed *A. capillus-veneris* as an experimental system. They not only systematically described the morphological process of this plant (Li et al., 2013), but established a culture system for shoot regeneration (Li et al., 2017). In the assay of gene expression during shoot regeneration from sporophyte tissue, they found that expression of *AcLEC1* was not only induced by stresses such as drought and ABA (Xie et al., 2008), but also by cultural conditions (Li et al., 2017). These observations sparked a modified hypothesis about the relationship between the origin of the seed maturation process and the *LEC1* gene: LEC1-type *HAP3* genes may origin for other functions, as it was induced upon stresses. However, if this gene expression was induced during embryogenesis, it may be endowed a novel function to be a master regulator for a seed maturation process.

The best way to test the hypothesis is to ectopically express *LEC1* gene during embryogenesis or archegonia development in pteridophytes to examine if the *LEC1* gene can trigger the seed maturation process, i.e., accumulation of storage products, acquisition of desiccation tolerance, and induction of dormancy (Harada, 1997). Unfortunately, gene transformation system of *A. capillus-veneris* has not yet been established. However, the induction of *AcLEC1* expression during tissue culture of sporophyte (Li et al., 2017) suggests that other approach can be used to test the hypothesis. While the prothalli cannot tolerant drought or ABA treatment, sugar might be the best candidate as an inducer of *AcLEC1* expression. The rationale of using sugar to induce *AcLEC1* expression during archegonia development underlies not only that the sugar is an important component in tissue culture in the MS media, but also the reports that sugar exerts its effect through induction of *LEC1* gene expression during drought response (Gupta and Kaur, 2005; Eveland and Jackson, 2012; Poonam et al., 2016) and more specifically affects genes involved in seed maturation including *LEC1* (Tsukagoshi et al., 2007).

Here, we firstly examined the expression pattern of *AcLEC1* during the entire life cycle of *A. capillus-veneris* and confirmed that no *AcLEC1* gene expression was detectable in prothallus. Afterward, we demonstrated that *AcLEC1* expression can be induced by both sucrose and glucose. Finally, as expected, we found that sugar treatments delayed prothallus development, promoted differentiation of reproduction organs, triggered accumulation of storage products. These findings demonstrated links between the sugar treatments and the induction of *AcLEC1* expression, as well as the sugar treatments and the events such as accumulation of storage products, which is similar to those considered as seed maturation process in seed plants. These links are supportive to the above mentioned modified hypothesis about the role of *LEC1* gene in origin of seed maturation process.

## Materials and Methods

### Plant Growth and Cultivation Conditions

Adult *A. capillus-veneris* plants were cultivated in greenhouses at Peking University (Beijing, China). Spores were collected and cultivated into cordate prothalli in sugar-free Knop’s agar medium as described (Li et al., 2013). For the sugar treatment, cordate prothalli were picked up with sterile hypodermic needles under anatomical lens and placed on Knop’s agar medium containing various sugar concentrations. When both antheridia and archegonia appeared, prothalli were sprinkled with sterile water to create appropriate conditions for fertilization. All prothallus cultivation experiments were conducted in a clean bench.

### Quantitative Reverse Transcription-PCR

Total RNA from all samples was extracted with the PureLink^TM^ Plant RNA Reagent (Invitrogen, Carlsbad, CA, USA) according to the manufacturer’s protocol. The total RNA samples were then treated with RQ1 RNase-Free DNase (Promega, Madison, WI, USA) to remove DNA contaminants. Each sample was reverse-transcribed into cDNA using the SuperScript First-Strand System for RT-PCR (Invitrogen, Carlsbad, CA, USA) following the manufacturer’s protocol. The quality of the RNA and cDNA was assessed by agarose gel electrophoresis. The RNA concentration was determined using a NanoDrop ND 1000 Spectrophotometer (Nano-Drop, Wilmington, DE, USA). qRT-PCR was performed with a 7500 Real-Time PCR System (Applied Biosystems, USA). The amplification reaction was carried out in a total volume of 20 μL, with 0.5 μL of each primer (10 μM), 1 μL of cDNA, 10 μL of Power SYBR Green I Master Mix kit (Bio-Rad, Hercules, CA, USA), and 8 μL RNase-free water. The PCR program was as follows: denaturing at 95°C for 10 min, followed by 40 two-step cycles (95°C for 15 s and 60°C for 64 s) and a final extension at 72°C for 5 min. Relative quantification of each gene was performed using the comparative threshold cycle method as described by Livak and Schmittgen (2001). It is reported that *AcCRYPTOCHROME GENE 2* (*AcCRY2*) stayed at the same level through the haploid and diploid phases (Imaizumi et al., 2000), thus we used *AcCRY2* as the internal control. Each sample was quantified at least in triplicate. The primer sets for each gene are listed in Supplementary Table 1.

### *In situ* Hybridization

Prothalli and pinnae at various stages were fixed in 4% paraformaldehyde (PFA) overnight at 4°C. After fixation, tissue samples were washed, dehydrated, and embedded in wax for sectioning and *in situ* hybridization as described by Zhang et al. (2013). *AcLEC1*-specific regions were amplified with primer sets 5′-GAAGATAGCAGATGATGCCAAGG-3′ and 5′-ATGAATCCCCCCGATACTACTAA-3′ and transcribed *in vitro* as probes using the Digoxigenin RNA labeling kit (Roche, Mannhein, Germany).

### Transient Sugar Treatment

Based on a report of sugar-dependent *LEC1* expression in *A. thaliana* (Tsukagoshi et al., 2007), *A. capillus-veneris* prothalli were treated transiently with sucrose or glucose. Cordate prothalli were immersed in buffer containing 30 g/L glucose for 30 min. Sugar-free buffer served as control. After rinsing in water, the treated cordate prothalli were transferred to a sugar-free medium. Prothalli were harvested 30 min, 1, 2 h, 1, 2, and 6 days after treatment followed by qRT-PCR to determine *AcLEC1* expression levels.

### Persist Sugar Treatment

Under sterile conditions, cordate prothalli growing on sugar-free medium were picked and transferred to medium containing 30 g/L glucose or 30 g/L sucrose. Sugar-free medium served as the control treatment. Prothalli were harvested 5, 10, 15, 20, and 25 days after transfer (DAT) for examination of *AcLEC1* gene expression by qRT-PCR.

### SEM Observation

The dissected samples were dehydrated, followed by submersion in a series of gradient alcohol-isoamyl acetate solutions as described previously (Li et al., 2013). Subsequently, the samples were dried by critical-point drying in CO_2_ (Hitachi HCP-2) for 6 h, mounted, and sputter-coated with gold/palladium (Hitachi E-1010). All samples were viewed under a Hitachi S-4800 SEM at 10.0 kV.

### Density and Size of Antheridia and Archegonia Analysis

Fertilized prothalli with apparently swollen archegonia were selected as samples. This method ensured that every prothallus sample was under similar developmental stage. For each treatment group, five fertilized prothalli were selected as samples and were observed under SEM. For each sample, three 25-mm^2^ area were randomly picked, in which the number of antheridia and archegonia were counted and the density was calculated. The diameter of mature archegonia or antheridia on the prothallus was measured by taking plotting scale as reference.

### Cytochemical Stain Assay

The control and sugar-treated prothalli were harvested 10, 20, and 30 DAT, dehydrated in a series of ethanol solutions, followed by exposure to a series of alcohol-acetone solutions as describe (Li et al., 2013). Next, the tissue samples were exposed to a series of acetone-Spurr’s resin solutions (acetone:resin ratio: 2:1, 1:1, 1:2, 0:1, 0:1, and 0:1), which were replaced every 8 h. Finally, the samples were embedded in Spurr’s resin. Sections (3 μm thick) were cut using a microtome (Leitz 1512, Germany) as described previously (Hu and Xu, 1990; Li et al., 2013). The periodic acid-Schiff (PAS) staining, Sudan black B staining, and Coomassie brilliant blue staining were chosen to label polysaccharides, lipids, and protein bodies, respectively, as described by Hu and Xu (1990). Images were captured with a light microscope (Zeiss Axioskop 2 Plus, Germany) using Axioplan software.

### *A. capillus-veneris* Storage Products Accumulation Related Gene Identification

In *Arabidopsis*, 184 seed-specific genes, excluding 30 genes that encode transcription factors, are known to be involved in storage products accumulation (Le et al., 2010). We screened the datasets of seed-specific genes (Mu et al., 2008; Le et al., 2010) and selected the genes involved in storage products accumulation by gene annotation. After gaining the gene candidates, we screened the *A. capillus-veneris* expressed sequence tags (ESTs) database by BLAST to find out the high identities ESTs of *A. capillus-veneris*. The genes with identities higher than 50% were considered to be storage products accumulation related genes in *A. capillus-veneris*. The three high identities genes were listed in Supplementary Table 2.

## Results

### Standardization of Developmental Stages of *A. capillus-veneris*

**FIGURE 1:**
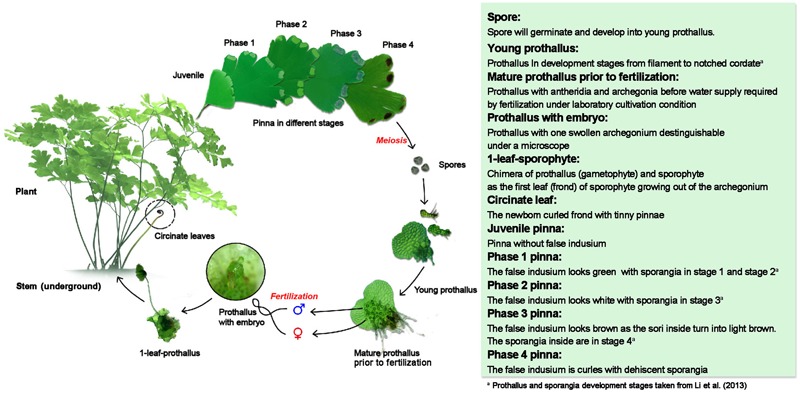
**Development stages of *Adiantum capillus-veneris*.** Schematic diagram in left part represents the 11 development stages during life cycle of *A. capillus-veneris.* Major morphological features are described in the right green box.

Previously, Li et al. (2013) have described the entire life cycle of *A. capillus-veneris* under cultivate conditions on the duration and morphogenetic characteristics. To clarify the expression pattern of *AcLEC1* during the life cycle of *A. capillus-veneris*, it is necessary to further divide the life cycle into development stages with distinguishable morphological characteristics for unambiguous sample collection. Based on the morphogenetic features, we divided the life cycle of *A. capillus-veneris* into 11 stages (**Figure 1**).

It needs to be mentioned that the above dividing system for developmental stages was mainly designed for the convenience of clarifying the expression pattern of *AcLEC1*. In general, it is more reasonable to set the zygote as a start point to describe a life cycle (Bai, 1999, 2015a,b, 2016, 2017). However, for the convenience of experimental operation, here we used spores as a start point. It should be better to adjust the start point into zygote if it becomes more accessible along with technology development. Another pragmatic consideration is that the above dividing system did not further divide prothallus into more stages although several morphologically distinguishable stages can be easily identified, such as club-shaped, early heart-shaped and so on (Li et al., 2013). Such a simplification mainly because of the growth condition for these stages are similar in terms of their effects on *AcLEC1* expression.

### *AcLEC1* Is Expressed in the Aerial Tissues of *A. capillus-veneris*

**FIGURE 2:**
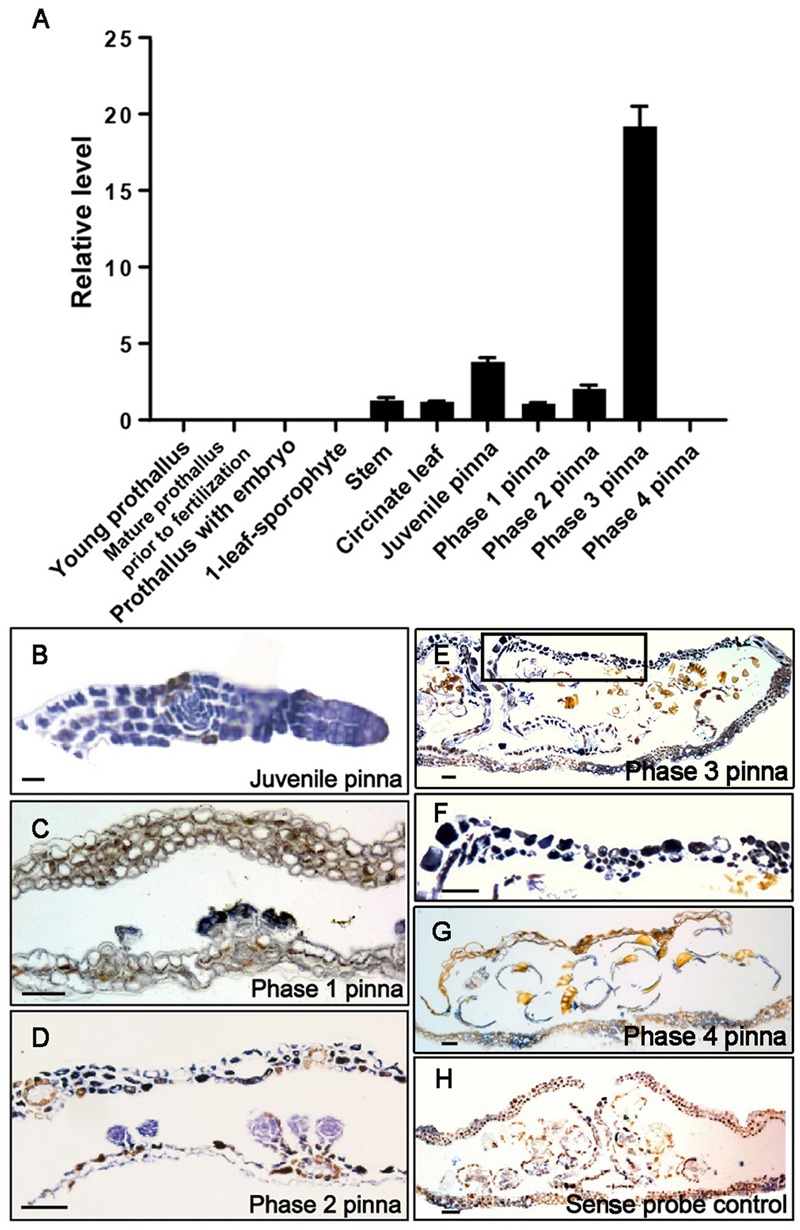
**The expression pattern of *AcLEC1*. (A)** *AcLEC1* expression level in 10 development stages and stem. The samples are collected according to **Figure 1**. At least four independent *A. capillus-veneris* lines were analyzed by quantitative reverse transcription-PCR (qRT-PCR) and results of three representative lines are shown. *AcCRY2* was used as an internal control. Results represent the average from three independent isolations of RNA ± SD. **(B–H)** *In situ* localization of *AcLEC1* transcripts in pinnae. The development stages of pinna proceed from **(B–E)** and **(G)** are indicated and **(F)** is the magnified image of black frame in **(E)**. Sense probe was used as a negative control **(H)**. Bar = 50 μm.

To test the hypothesis that the specific spatiotemporal pattern of LEC1-type *HAP3* gene expression is critical for proper execution of the seed maturation process, we examined the expression pattern of *AcLEC1* in *A. capillus-veneris.* Samples of 10 stages during the entire life cycle and stem were collected according to **Figure 1**. Quantitative reverse transcription-PCR (qRT-PCR) was employed to determine *AcLEC1* expression levels. **Figure 2A** shows that *AcLEC1* mRNA was rarely detectable in the prothallus samples and 1-leaf-sporophyte samples. In contrast, *AcLEC1* mRNA was detected at various levels in the stem and pinna at various stages. The highest expression level was detected in the sample of phase 3 pinna where sporangia forms (**Figures 1, 2A**). Such expression pattern is consistent to previous finding that *LEC1* genes are expressed in aerial tissues and at high level in sporangia (Kirkbride et al., 2013).

To further examine the preference of *AcLEC1* expression in difference tissues, *in situ* hybridization was carried out. Signal of *AcLEC1* mRNA was detected from juvenile to phase 3 pinna (**Figures 2B–F**). Consistent to the highest expression level detected with qRT-PCR, the *in situ* hybridization confirmed the highest signals in phase 3 pinna (**Figures 2E,F**). It is interesting that no *AcLEC1* mRNA is detected after spore release in phase 4 pinna (**Figures 2A,G**).

### *AcLEC1* Expression Is Sugar-inducible during Prothallus Development

The above data demonstrated that under normal growth conditions, there is no *AcLEC1* expression in prothallus development (**Figure 2A**). Therefore, we can use prothalli to examine whether sugar can induce *AcLEC1* expression.

**FIGURE 3:**
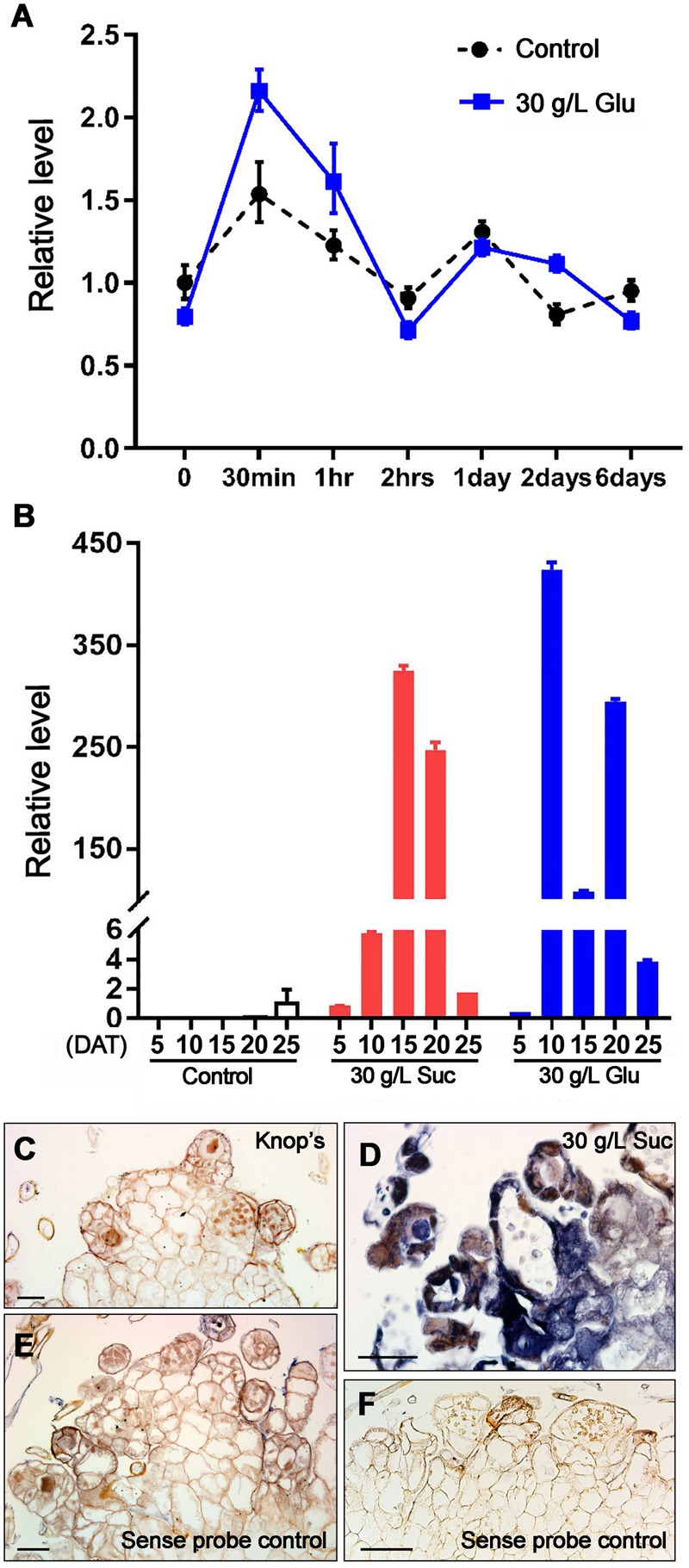
***AcLEC1* is sugar-inducible. (A)** qRT-PCR result of *AcLEC1* expression levels in prothalli which respectively grown for 0–6 days after transient (DAT) treatment with either 30 g/L glucose (blue line) or sugar-free buffer (black dotted line). **(B)** qRT-PCR result of *AcLEC1* expression levels in prothalli which grown for 5, 10, 15, 20, or 25 DAT to medium containing sucrose (red columns) or glucose (blue columns). Control stands for Knop’s medium (blank columns). In **(A,B)**, *AcCRY2* was used as an internal reference. Results represents the average from three independent isolations of RNA ± SD. **(C–F)** *In situ* localization of *AcLEC1* transcripts in prothalli. Mature prothalli transferred to Knop’s medium **(C)** or medium containing 30 g/L sucrose **(D)** were detected by anti-sense probe of *AcLEC1*. **(E,F)** Negative control using sense probe for samples in **(C,D)**, respectively. Bar = 50 μm.

In our pilot experiments, we found 20 and 30 g/L sugar can induce *AcLEC1* expression but 50 g/L can inhibit prothalli growth. So we used concentration of 30 g/L as a treatment. Firstly, we examined whether the *AcLEC1* expression can be transiently induced. **Figure 3A** shows that *AcLEC1* expression can be induced upon 30 min treatment, higher than the control. Such expression is dependent on existence of the sugar (Glucose here), as the expression decrease upon withdraw of the sugar.

To observe the effects of sugar treatments on prothallus development, we further examined the *AcLEC1* expression in prothallus development under continuous sugar-medium culture. Considering the developmental process from spore germination to cordate prothallus taking 20 days and from cordate prothallus to fertilization taking about 25 days (Li et al., 2013), here we examined *AcLEC1* expression every 5 days starting from the cordate prothallus culture. **Figure 3B** shows that *AcLEC1* gene expression was significantly induced after 10-days culture on the sugar media, both sucrose and glucose. Interestingly the expression level on sucrose medium showed one peak at 15-days, but that on glucose medium showed two peaks, one at 10-days culture and another at 20-days culture.

To verify the *AcLEC1* expression during prothallus development, *in situ* hybridization was carried out. Strong signals of *AcLEC1* probe were detected in prothalli, especially in archegonia and antheridia (**Figures 3C–F**).

### Sugar Treatments Inhibit Prothallus Development

**FIGURE 4:**
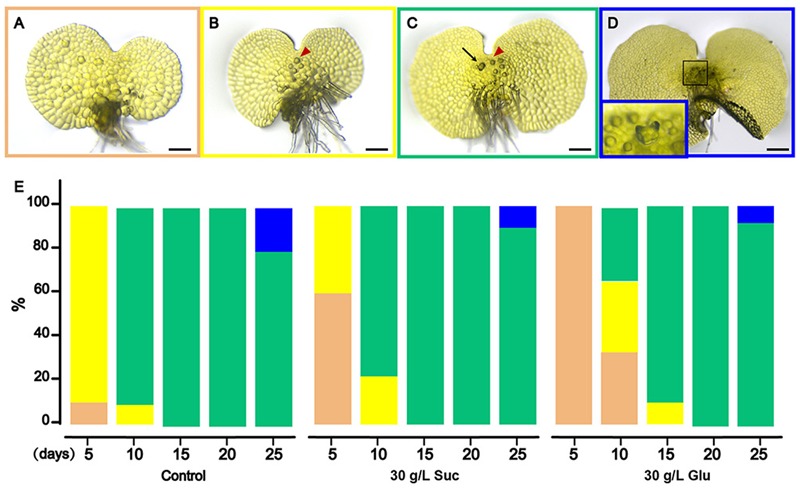
**Sugar treatment inhibits prothallus development. (A–D)** The bright field images of prothalli in four development stages monitored in this research: the cordate prothallus **(A)**, antheridia initiation **(B)**, archegonia initiation **(C)**, and embryogenesis **(D)**. The embryogenesis is happened in the swollen archegonium indicated by a black frame and magnified in the embedded inset at the bottom left corner. Red arrowheads point to the antheridium. Black arrow points to the archegonium. **(E)** Percentage of correspondent differentiation status. The colors of columns in **(E)** are corresponding to the same colors in **(A–D)**, respectively. Bar = 200 μm.

To examine the effects of sugar treatments on prothallus development, we analyzed the differentiation status of prothallus development. As described in Li et al. (2013), prothallus development goes through filament, clavate, broadened and cordate stages after spore germination. Following cordate stage, differentiation of prothallus mainly exhibits as initiation and differentiation of antheridia and archegonia (Li et al., 2013). As the prothalli were cultured from young cordate stage, we monitored the rate of initiation of antheridia and archegonia under sugar treatments. **Figures 4A–D** show typical differentiation status of prothalli, in which A shows the cordate prothallus without antheridium or archegonium initiation; B shows antheridium initiation (red arrowhead pointed); C shows both antheridium (pointed by red arrowhead) and archegonium initiation (pointed by black arrow); and D shows opened archegonia with embryogenesis (magnified in the inset). For convenience, each status from A to D framed with different colors.

Using the above morphological criteria, percentage of correspondent differentiation status in all the examined prothalli were counted (**Figure 4E**). On control medium, majority of cultured prothalli entered the stage B (antheridium initiation) in 5 DAT. In 15 DAT, all prothalli entered the stage C. In comparison, the differentiation process slowed down on 30 g/L sucrose medium, indicated by larger proportions of 5 DAT prothalli retaining at stage A and 10 DAT prothalli retaining at stage B. The differentiation of prothalli was more severely postponed on the 30 g/L glucose medium.

### Sugar Treatments Promote Formation of Reproductive Organs

**FIGURE 5:**
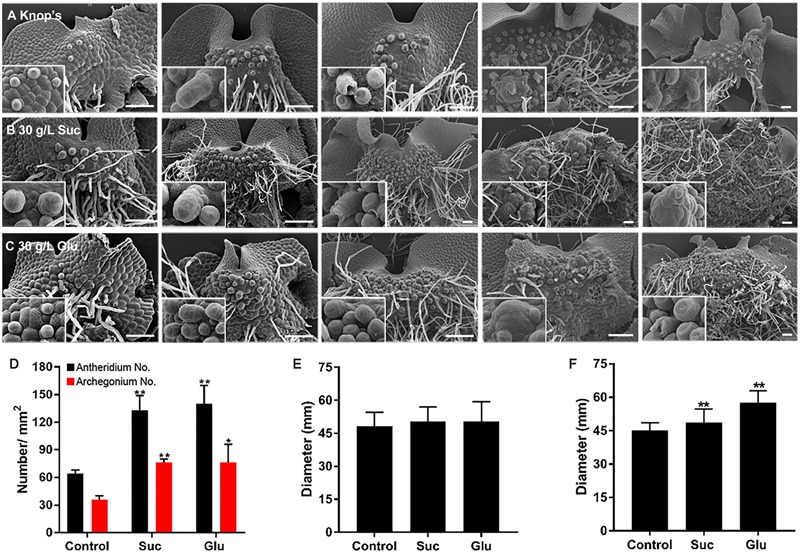
**Sugar treatments promote formation of reproductive organs. (A–C)** SEM images of prothalli on Knop’s medium **(A)**, 30 g/L sucrose medium **(B)** or 30 g/L glucose medium **(C)** at 10, 20, 30, 40, and 50 DAT. Higher magnification images of reproductive organs or distinguishable embryo were shown in embedded insets. **(D)** The density of antheridium (black columns) and archegonium (red columns) in fertilized prothalli. The diameter of archegonium **(E)** and antheridium **(F)** of fertilized prothalli with or without sugar treatment. Data represents means of five independent samples ± SE. Asterisks above the bars report the results of a significance test (Student’s *t*-test) for differences between the control and the treated samples: ^∗∗^*P* < 0.01, ^∗^*P* < 0.5. Bar = 200 μm.

While the differentiation rate was decreased upon the sugar treatment, the densities of antheridia and archegonia were increased. Based on SEM observation, we can count the number of antheridia and archegonia for density calculation. **Figures 5A–C** (five panels in each lane represent the differentiation status of antheridia and archegonia at 10, 20, 30, 40, and 50 DAT) shows that comparing to the sugar-free Knop’s culture, density of both antheridia and archegonia increases. The quantitation of the densities at the fertilization stage (40 DAT) were shown in **Figure 5D**.

In addition, although no distinguishable alteration in architecture of antheridium nor archegonium were found, the size of archegonia and antheridia were increased, significantly for antheridia (**Figures 5E,F** respectively).

### Sugar Treatments Trigger Accumulation of Storage Products

Among the three physiological and biochemical processes consisting the seed maturation process, the accumulation of storage products is the most prominent characteristic. To examine whether the sugar treatments can trigger the accumulation of storage products, we analyzed the accumulation of representative storage macromolecules, e.g., polysaccharides, proteins, and lipids during prothalli development by histochemical stain.

**FIGURE 6:**
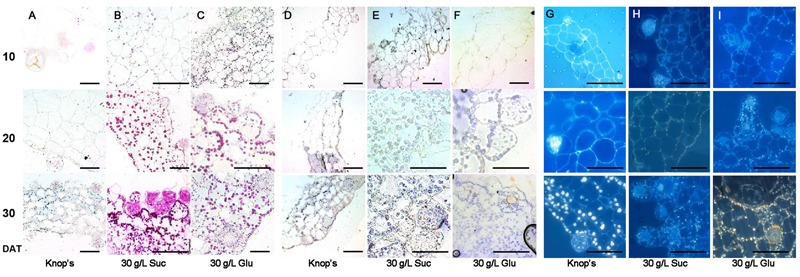
**Sugar treatment triggers accumulation of storage products.** Prothalli were persistently treated by 30 g/L sucrose or glucose. 10, 20, and 30 DAT prothalli were labeled by cytochemical stain technique. The images of representative sections under the same treatment during 10–30 DAT were arranged in one column. **(A–C)** Cherry red indicates the polysaccharides which labeled by PAS reaction. **(D–F)** Blue display the protein body after Coomassie brilliant blue staining. **(G–I)** The lipid bodies were manifested by gray-blue using Sudan black B staining. Bar = 50 μm.

**Figures 6A–C** show the accumulation of polysaccharides with PAS stain during prothalli development at three different stages (10, 20, and 30 DAT), and on three different mediums, i.e., the sugar-free Knop’s (as control), 30 g/L sucrose and 30 g/L glucose. It is obvious that no PAS signals were detected at 10 DAT prothalli on all three mediums. However, after 20 DAT, obvious PAS signals were detected on the sugar-treated prothalli, not on the control prothalli. The strongest staining was detected on the prothalli at 30 DAT on the sucrose medium (**Figure 6B**, 30 DAT). This suggests the unambiguous accumulation of polysaccharides in the prothalli under sugar treatment.

**Figures 6D–F** show the accumulation of protein with Coomassie brilliant blue stain during prothalli cultured under sugar treatments. While not that obvious signals detected for storage protein as did for starch in prothalli under sugar treatments, the signals (stained to blue) were more intensive in sugar-treated prothalli than control.

**Figures 6G–I** show the accumulation of lipid with Sudan black B stain. The typical lipid signal by Sudan black B stain should be gray-blue and the background should be sky blue or blue (Supplementary Figure 1A). The signals in sugar-treated prothalli is much intensive than control (Supplementary Figures 1B,C).

### Sugar Treatments Induce Expression of Homologs of *Arabidopsis* Seed Genes

To further explore whether the accumulation of storage products in prothalli grown on sugar mediums is similar to that in seed maturation process, we carried out a molecular analysis.

Firstly, we used three *Arabidopsis* seed-specific genes, *SUCROSE-PHOSPHATE SYNTHASE* (*SPS*), *CRUCIFERIN2* (*CRU2*), and *FATTY ACID ELONGASE1* (*FAE1*), as queries to screen *A. capillus-veneris* homologs from NCBI EST database. These three genes are respectively involved in accumulation of starch (*SPS*), storage protein (*CRU2*) and lipid (*FAE1*). The ESTs homologous in *A. capillus-veneris* to these three genes were therefore designated as *AcSPS, AcCRU2*, and *AcFAE1*, respectively. Afterward, we analyzed the expression pattern during prothallus development.

**FIGURE 7:**
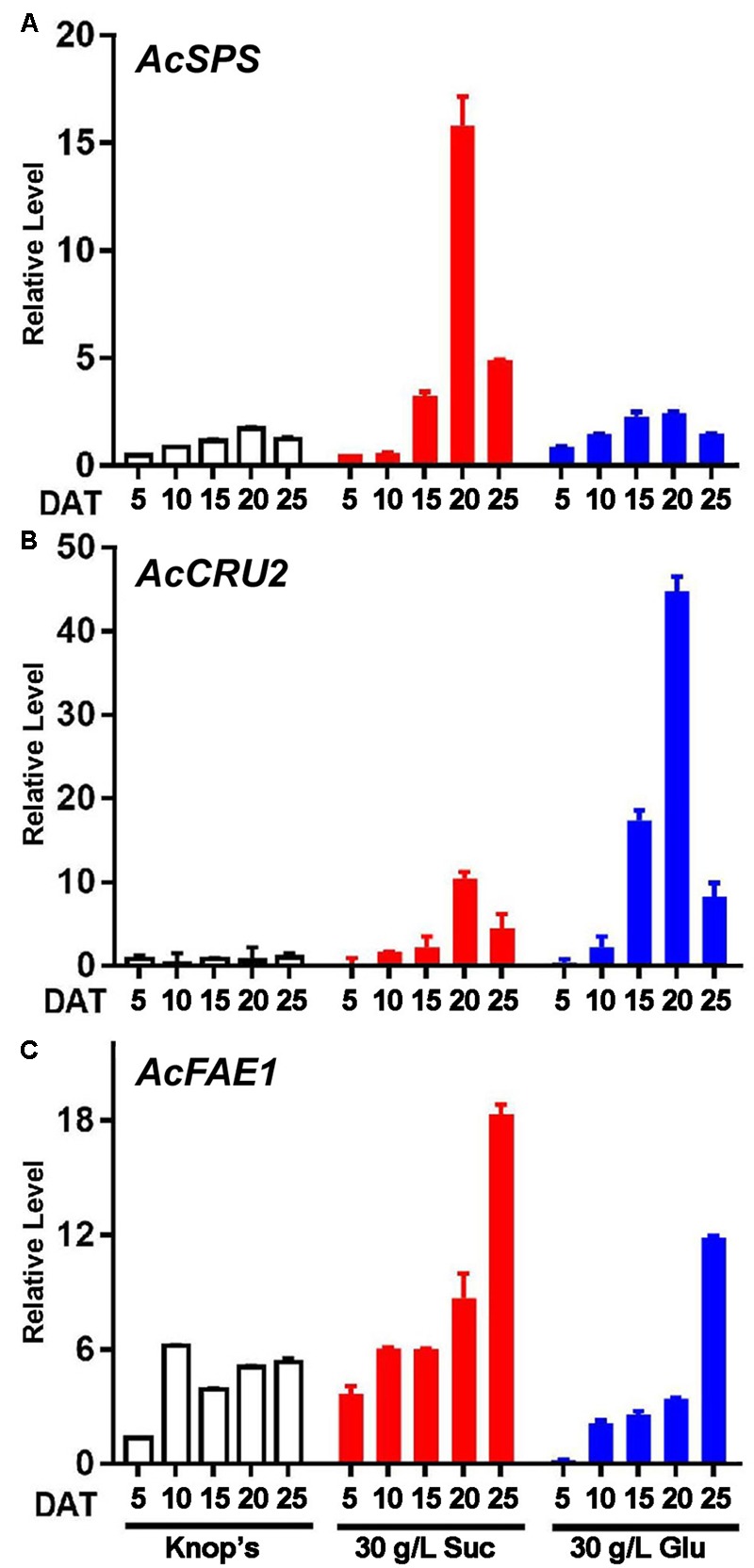
**Sugar treatment induces expression of *Arabidopsis* seed homologs.** qRT-PCR examine results of **(A)** *AcSPS*, **(B)** *AcCRU2*, and **(C)** *AcFAE1*. *AcCRY* was used as an internal reference. Results represents the average from three independent isolations of RNA ± SD.

**Figure 7** shows that consistent to the accumulation of storage macromolecules in the sugar-treated prothalli, the *AcSPS* expression was significantly induced in the prothalli on the sucrose medium at 20 DAT (**Figure 7A**). The induction of *AcCRU2* is highest in the prothalli on the glucose medium at 20 DAT (**Figure 7B**). High induction of *AcFAE1* was also observed in the prothalli on the sucrose medium, but delayed to 25 DAT (**Figure 7C**).

## Discussion

Based on the current knowledge about the role of *LEC1* gene in seed maturation process, we proposed a modified hypothesis that the process called seed maturation identified in seed plants may be triggered by the induction of *LEC1* gene expression during embryogenesis in non-seed plants. To test the hypothesis, we firstly confirmed that there is no detectable expression of *AcLEC1*, a homolog to *Arabidopsis LEC1* in a non-seed plants *A. capillus-veneris*, during prothallus development, where the embryogenesis occurs in archegonia (**Figure 2**). Then, we demonstrated that *AcLEC1* expression can be induced by sugar treatments during prothallus development (**Figure 3**). In parallel, we found that the sugar treatment can trigger accumulation of storage products (**Figure 6**), one of the hallmark events in seed maturation process during prothallus development; promote differentiation of reproductive organs (**Figure 5**), i.e., antheridia and archegonia; and delay the differentiation of prothalli (**Figure 4**), the effect similar to another hallmark event, dormancy. Consistent to the accumulation of storage products, we found that the genes homologous to so called seed-specific genes were activated in sugar-treated prothalli (**Figure 7**). While these findings indicate the links between sugar treatments and *AcLEC1* expression, as well as sugar treatments and events such as accmulation of storage products, which is occurring in prothalli, mimicking to that called seed maturation process, what would these findings imply to the origin of the seed maturation process?

Firstly, the property of inductive expression *AcLEC1* imply its potential to be coopted into the origin of seed maturation process. Previous investigation already demonstrated that in *Arabidopsis, LEC1* expression can be induced in the *KK* mutant at seedling stage (Tsukagoshi et al., 2007). Xie et al. (2008) and Li et al. (2017) also demonstrated that *AcLEC1* can be induced under drought and ABA treatment of sporophyte and tissue culture respectively. It seems that the *LEC1*-like gene was not originated to be a key regulator of seed maturation process as it does not express during embryogenesis of non-seed plants, rather a stress response gene as it expressed in aerial tissue as we demonstrated in **Figure 2** and its expression can be induced by drought and ABA (Xie et al., 2008). Such an inducibility makes it possible that the expression of *LEC1*-like gene can be induced in nature by chance during embryogenesis as we found in this work in lab by intention. From this perspective, our findings in this work open up a new window to investigate origin of seed maturation process by further investigation of molecular mechanism of induction of *LEC1*-like gene expression in non-seed plants.

Secondly, if the accumulation of storage products is a hallmark event of seed maturation process, it would be interesting to ask, whether such a process is seed specific or not. According to Harada (1997), the seed maturation process is intrusive to the embryogenesis, implying that it is an independently originated process of embryogenesis. The accumulation of storage products can be found in other tissues such as tuberous roots in cassava and sweet potato and tubers in potato. From this perspective, the accumulation of storage products should not be specific to the structure called seed. While our findings revealed the sugar treatments can trigger the accumulation of storage products during prothallus development, it would be interesting ask a reverse question that why there is no obvious accumulation of storage products in prothallus development in nature?

If we consider the stress inductive property of *LEC1* expression and sugar induction of both *AcLEC1* expression and accumulation of storage products in prothalli, it will be interesting to hypothesize that the reasons for no accumulation of storage products come from lack of stress conditions, as the prothalli grown in wet habitation, and/or lack of additional assimilate supply for storage products to be synthesis. Such a hypothesis is mutually complementary to the current theory on origin of seeds. Current theory suggests that the seed is originated from the terminally located ovule where the embryogenesis occurs (Taylor and Taylor, 1993; Herr, 1995; Niklas, 1997; Doyle, 2006). According to the telome theory, the terminally grown ovules are obvious the aerial grown tissues. If our findings are generally applicable, the *LEC1*-like genes should be expressed in the aerial grown ovules. On the other hand, according to the “source-sink” theory of assimilation allocation (Rolland et al., 2006; Eveland and Jackson, 2012), the terminally located ovules should function as a sink in an assimilation flow. If it is the case, the imported assimilates, similar to the sugar treatments in our experiments, can trigger jointly by enhancing the expression of *LEC1*-like genes, the accumulation of storage products in the terminally localized ovules and superposed upon embryogenesis occurring in the ovules, and therefore a novel structure emerged, latterly called seed. Once it happened, no force can prevent such a trait been selected during evolution for the obvious adaptive advantages. With this hypothesis in mind, the mechanism of origin of seed maturation process, or briefly called seed program, can be empirically investigated, and the exploration of origin of seed would be no longer the privilege of paleobotanists.

Finally, although it is demonstrated that *LEC1* gene is a key regulator in seed maturation process or seed program, because of the lack of tools of gene transformation, it is not yet clear whether the effects on prothallus development of sugar treatments come directly from the sugar-induced *AcLEC1* expression or other mechanisms. Even though, the findings in this work paved a road to the prosperous future to uncover the secret on origin of the seed.

## Author Contributions

Y-HF designed the experiments, conducted the experiments and drafted the manuscript. XL designed the experiments, conducted cytochemical stain assay and time course assay, and revised the manuscript. S-NB and G-YR conceived the study, reviewed and edited the manuscript. All authors read and approved the manuscript.

## Conflict of Interest Statement

The authors declare that the research was conducted in the absence of any commercial or financial relationships that could be construed as a potential conflict of interest.
