# Supplementary material for: Sugar Treatments Can Induce AcLEAFY COTYLEDON1 Expression and Trigger the Accumulation of Storage Products during Prothallus Development of Adiantum capillus-veneris
Source: Front Plant Sci. 2017 Apr 21;8:541. doi: 10.3389/fpls.2017.00541 (PMC5399092; doi:10.3389/fpls.2017.00541)
Supplement: Supplementary file 3 [file Data_Sheet_1.docx]

Supplementary Material

Sugar treatments can induce *AcLEAFY COTYLEDON1* expression and trigger the seed maturation process during gametophyte development of *Adiantum capillus-veneris*

Yu-Han Fang, Xia Li, Shu-Nong Bai^*^, Guang-Yuan Rao

***Correspondence:**

Prof. Shu-Nong Bai: shunongb@pku.edu.cn;

# Supplementary Data List

Supplementary Table 1: Primer sets for qRT-PCR

Supplementary Table 2: Similarity analysis of storage reserves accumulation related genes between *A. capillus-veneris* and *A. thaliana*

Supplementary Figure 1: Signals in Sudan black B stain assay

# Supplementary Figure 1


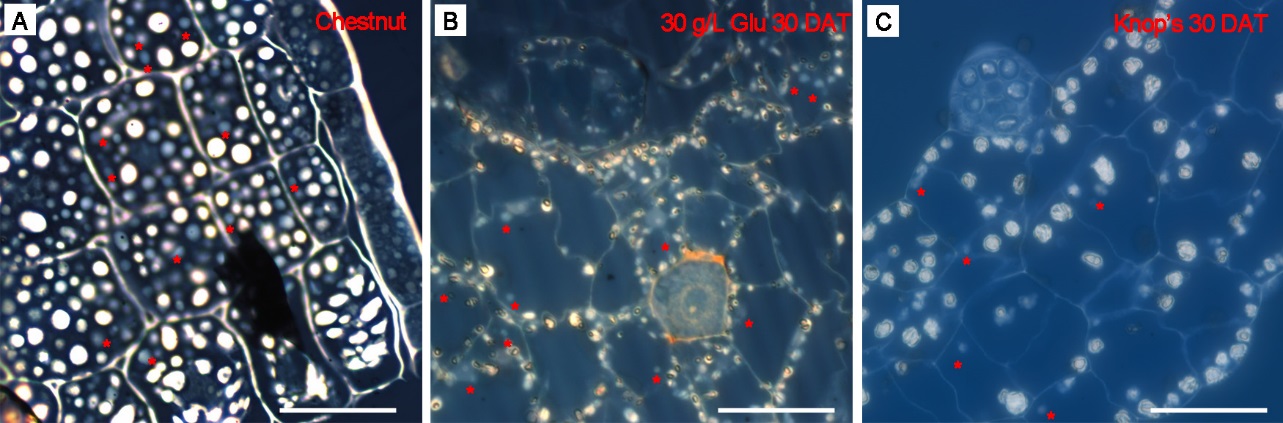


**Supplementary figure 1.** Signals in Sudan black B stain assay. **(A)** Chestnut used as positive control to indicate the gray-blue (above red asterisk, the same below) and the blue background. **(B-C)** Signals in sugar-treated prothallus **(B)** is much intensive than control **(C)**. Bar = 50 μm.
